# Astaxanthin-Rich *Haematococcus pluvialis* Algal Hepatic Modulation in D-Galactose-Induced Aging in Rats: Role of Nrf2

**DOI:** 10.15171/apb.2018.061

**Published:** 2018-08-29

**Authors:** Farouk Kamel El-Baz, Rehab Ali Hussein, Gehad Abdel Raheem Abdel Jaleel, Dalia Osama Saleh

**Affiliations:** ^1^Plant Biochemistry Department, National Research Centre, Giza, Egypt.; ^2^Pharmacognosy Department, National Research Centre, Giza, Egypt.; ^3^Pharmacology Department, National Research Centre, Giza, Egypt.

**Keywords:** Hepatic modulation, D-galactose, Aging, Haematococcus pluvialis, Astaxanthin

## Abstract

***Purpose:*** Aging is associated with hepatic morphological and physiological deterioration due to the accumulation of endogenous and exogenous free radicals and the resultant oxidative stress. The present study aims to investigate the effect of Haematococcus pluvialis microalgae on hepatic changes associated with D-galactose (D-Gal)-induced aging in rats.

***Methods:*** Aging was induced in rats by daily intraperitoneal injection of D-Gal (200 mg/kg/day) for eight consecutive weeks. D-Gal-injected rats were treated by astaxanthin (ATX)-rich H. pluvialis biomass, its carotenoid and polar fractions for two weeks. Twenty four hours after the last dose, blood samples were collected and the liver tissues were isolated for further biochemical and histopathological examinations.

***Results:*** D-Gal induced aging was associated with an elevation in serum liver function parameters, hepatic oxidative stress biomarkers viz., catalase (CAT), glutathione transferase (GST) and myeloperoxidase (MPO), as well as decreased expression of nuclear factor like-2 (Nrf2). Moreover, induction of aging exhibited an elevation of hepatic inflammatory cytokine; interleukin-6 (IL-6) and its modulator; nuclear factor Kappa B (NF-KB). However, treatment of D-Gal injected rats with ATX-rich H. pluvialis restored the serum liver function parameters as well as hepatic CAT, GST and MPO levels with an elevated expression of Nrf2. Treatment with ATX-rich H. pluvialis was also accompanied with a decrease in hepatic levels of NF-KB and IL-6. Histopathological examination emphasized all the previous results. Similarly, all trans-astaxanthin showed high affinity towards Nrf2 with -7.93 kcal/mol estimated free energy of binding as well as moderate affinities towards IL-6 and NF-KB through a docking study.

***Conclusion:*** ATX-rich H. pluvialis showed beneficial effects by ameliorating the hepatic changes associated with D-Gal induced aging in rats due to its modulatory role of the Nrf2/Keap pathway.

## Introduction

Aging is a spontaneous biological phenomenon of great medical importance which is associated with a marked decrease in antioxidant status in all vital organs. The liver is a resilient organ that maintains its homeostatic functions with age. Nevertheless, certain age-related changes do occur in the senescent liver that deserves consideration.^1^ Studies done to reveal the changes associated with liver aging proved that aging causes gradual alteration in hepatic function and structure and can increase risk factors to many hepatic diseases thus, increasing the mortality rate.^2^

Several studies were performed in seek of knowing the exact mechanism for aging. Strong evidence was provided that the main cause is increasing in the production of free radicals from mitochondria resulting in increasing oxidative stress and antioxidant defense mechanism.^3,4^ Oxidative stress cause hepatic damage by alternations of protein, lipid, and DNA components. Also, oxidative stress causes many diseases including: chronic liver failure, liver fibrosis, and aging.^5^

Nuclear Factor Like-2 (Nrf2); a key transcription factor that regulates the antioxidant defense mechanism; is essential for healthy regeneration and inhibition of chronic diseases.^5,6^ It is responsible for gene expression and production of protein products used in detoxification and elimination of reactive oxidants through conjugation reactions. Nrf2 activation is regulated by Keap-1 by binding to specific DNA which in turn produces extremely powerful antioxidants enzymes that is considered the most powerful action protecting the human body from oxidative stress state.^7^

Protection from aging-associated diseases can be done through two approaches either lifestyle modification or anti-aging supplementation.^8^ *Haematococcus pluvialis* is a single-cell Chlorophyte algal species, found worldwide and enters in many industries; nutriceuticals, pharmaceuticals, cosmetics and aquacultures. It was indicated that *H. pluvialis* species is well known for its high content of the strong antioxidant astaxanthin (ATX) that can be protective against several kinds of oxidative damage.^9,10^

The main objective of the present study is to investigate the ameliorative effect of *H. pluvialis* biomass, carotenoid and polar fractions on hepatic aging progression and to assess the probable underling mechanism. To achieve this aim, first, a docking study was performed to define the affinity of ATX towards inflammatory mediators interleukin-6 (Il-6) and nuclear factor kappa B (NF-KB) and transcription factor nuclear factor like-2 (Nrf2), followed by an *in vivo* study performed on D-Gal-induced aged rats.

## Materials and Methods

### Preparation, saponification and Estimation

#### Preparation of algal fractions

The dried biomass of *H. pluvialis* was obtained from Algal Technology Lab., NRC and was grinded thoroughly for cell wall disruption. The carotenoid fraction was prepared by solvent extraction using hexane, ethyl actetae (80:20) till complete exhaustion.^11^ The residue of the microalgal biomass was allowed to dry and further extracted with 70% methanol till complete exhaustion to render polar fraction. The dried fractions were kept in dark bottles at a temperature less than 4 °C for further analysis.

#### Saponification of carotenoid fraction:

Freshly prepared sodium hydroxide methanolic solution (5 %) was added to the carotenoid fraction solution in the ratio 1:5 (v/v). The hydrolysis reaction of ATX esters was carried out overnight in darkness at ambient temperature.

#### Estimation of free and conjugated astaxanthin:

The carotenoid fraction (1mg) before and after saponification were dissolved in 5 ml methanol/acetone (1:1), filtered through 0.45 mm membrane filter, and kept in the dark for the analysis of free and conjugated ATX, respectively. Standard solutions of ATX and β-carotene were prepared by dissolving 1mg of each of authentic ATX (purchased from Sigma-Aldrich, Germany) in 5ml methanol/acetone (1:1), ), filtered through 0.45 mm membrane filter, and kept in the dark.

Analytical HPLC was performed with a Zorbax-C18 column (5mm; 250 mm x 4.6 mm) on an Agilent 1200 series instrument equipped with an online diode-array detector. An isocratic elution was done using mobile solvent system: methanol/water/dichloromethane/acetonitrile (70:4:13:13, v/v/v/v). The analysis was carried out at a flow rate of 1.0 ml/min at room temperature. Chromatograms were recorded at 480 nm, and UV-vis absorption spectra were recorded online with the photodiode-array detection system.

### Docking study

Docking calculations were carried out using DockingServer.^12^ The MMFF94 force field was used for energy minimization of ligand molecule (Astaxanthin) using DockingServer. Gasteiger partial charges were added to the ligand atoms. Non-polar hydrogen atoms were merged, and rotatable bonds were defined. Docking calculations were carried out on NF-KB, IL-6 and Nrf2 protein models. Essential hydrogen atoms, Kollman united atom type charges, and solvation parameters were added with the aid of AutoDock tools.^13^ Affinity (grid) maps of 20×20×20 Å grid points and 0.375 Å spacing were generated using the Autogrid program. AutoDock parameter set- and distance-dependent dielectric functions were used in the calculation of the van der Waals and the electrostatic terms, respectively. Docking simulations were performed using the Lamarckian genetic algorithm (LGA) and the Solis & Wets local search method.^14^

### Pharmacological study

#### Animals

Male Westar albino rats weighing 130–150 g were obtained from the Animal House Colony of the National Research Centre. Animals were kept under standardized conditions (temperature 22 ± 1 °C, relative humidity 55 ± 15%, with a 12 h light and dark cycle and were allowed food and tap water ad libitum).

#### Chemicals 

D-Galactose (D-Gal) was purchased from Sigma–Aldrich (St. Louis, Missouri, USA). All other chemicals used were purchased from standard commercial suppliers and were of analytical grade quality.

#### Experimental design

Aging was induced in rats by subcutaneous injection with D-Gal (200 mg/kg/day) for eight consecutive weeks. Thirty albino rats were allocated into five groups, each group includes six rats. Group I served as a negative control group, group II served as positive control which received D-Gal while groups III, IV and V received D-Gal then injected with *H. pluvialis* biomass (BHP; 450 mg/kg; o.p.), its polar fraction (PHP; 30 mg/kg; p.o.) and carotenoid fraction (CHP; 30 mg/kg; p.o.), respectively for 2 weeks (fractions doses were calculated according to their yield). Twenty-four hours after the last dose of the *H. pluvialis* treatments, blood samples were collected, animals were sacrificed; liver was isolated.

#### Biochemical assessment:

Serum levels of aspartate aminotransferase (AST)^15^ and alanine aminotransferase (ALT)^15^ as well as the hepatic triglycerides (TG)^16^ and total cholesterol (TC)^17^ levels were measured colorimetrically. Hepatic levels of catalase,^18^ glutathione-S-transferase (GST),^19^ interleukin 6 (IL-6),^20^ cytokine modulator nuclear factor kappa (NF-kappa), nuclear factor like-2 (Nrf2) and myeloperoxidase (MPO) were also determined with ELISA kits according to the manufacture procedures.

#### Histopathological assessment:

Liver samples were dissected out, fixed in 10% formalin. The sections were taken at 5 μm thickness, stained with alum-haematoxylin and eosin and examined microscopically for the evaluation of histopathological changes.

#### Statistical analysis

Data are presented as mean ± SE. Statistical analysis of the data was carried out using one way analysis of variance (ANOVA) followed by Tukey’s multiple comparison test to judge the difference between the various groups. Statistical significance was acceptable to a level of P < 0.05. Data analysis was accomplished using the software program GraphPad Prism (version 5).

## Results and Discussion

**Figure 1:**
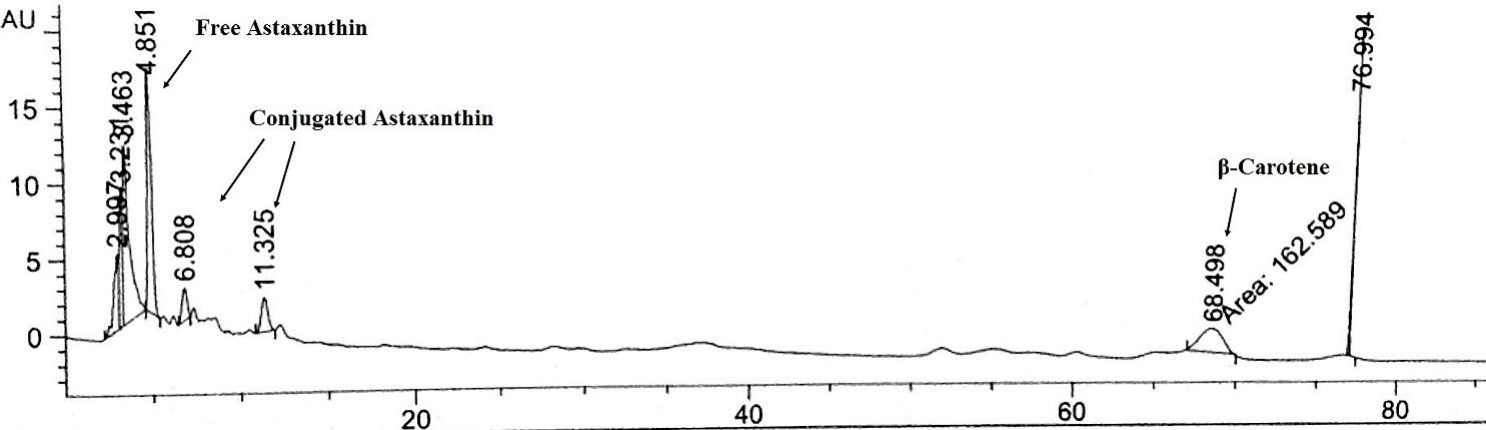
HPLC chromatogram of the carotenoid fraction of *H. pluvialis*

HPLC analysis of the carotenoid fraction of *H. pluvialis* led to the separation and identification of ATX and β-carotene as compared with standard samples run under the same conditions as illustrated in Figure 1. Quantitative analysis revealed the presence of 28.7 mg/100g free ATX and 15.5 mg/100g β-carotene. The analysis was repeated after the saponification of ATX esters and the chromatogram revealed the increment of the peak of ATX with the disappearance of other peaks which indicated the liberation of free ATX which was estimated to be 49.99 mg/100g with 21.2 mg/100g increase attributed to the amount of conjugated ATX. The carotenoid rich fraction of *H. pluvialis* was analyzed using LC-DAD/ESI-MS and the major carotenoids were identified either free as well as bounded to fatty acids in our previous work.^21^

In the present study, induction of aging in albino rats by D-Gal (200mg/kg I.P), a well-known model for aging^22^ for 8 consecutive weeks was associated with an elevation in sera liver function parameters such as ALT and AST and hepatic TG and TC. There are a lot of mechanisms of D-Gal inducing aging such as increased production of oxidants, cause changes in activity of antioxidants enzymes and may cause oxidative damage accumulation which led to alteration in catalase, GST and MPO. Moreover, D-Gal was accompanied with a prominent elevation of hepatic inflammatory cytokine; IL-6 and its modulator; NF-KB.

**Figure 2:**
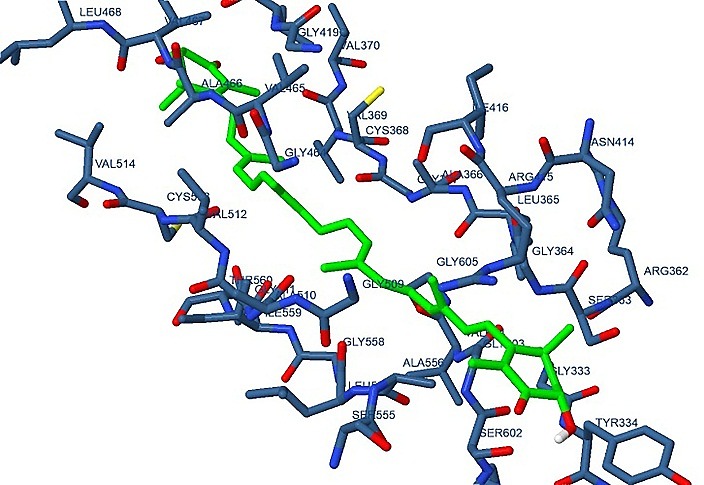
Docking of all-trans ATX on Nrf2

The docking of all trans-astaxanthin on IL-6, NF-KB and Nrf-2 showed that ATX possesses high affinity towards Nrf2 where the estimated free energy of binding was -7.93 kcal/mol as illustrated in Figure 2 as well as moderate affinities towards IL-6 and NF-KB, +1.86e+04 kcal/mol and +0.12 kcal/mol, respectively. The docking study was repeated for the 9-cis ATX and the estimated free energies of binding were +145.15, +1.16e+03 and -4.90 kcal/mol for Nrf2, IL-6 and NF-KB, respectively.

**Figure 3:**
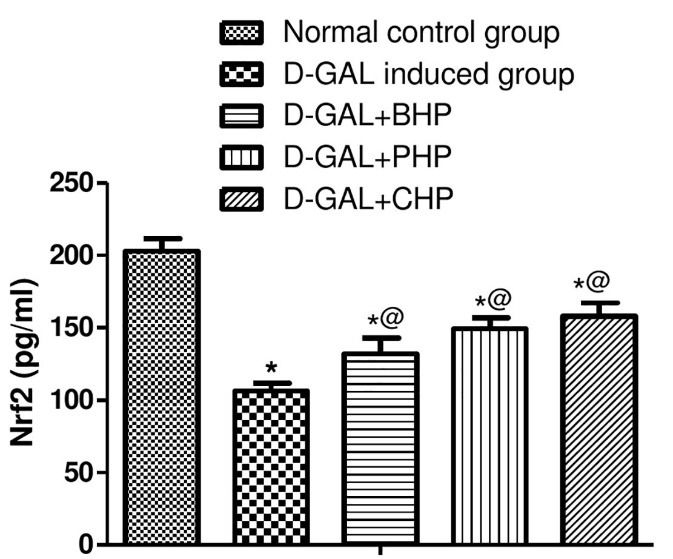
Effect of *H. pluvialis* on Nrf2 in D-Gal induced hepatic aging in albino rats
*: significantly different from normal control group at P≤0.05
@: significantly different from D-Gal-treated group at P≤0.05

**Figure 4:**
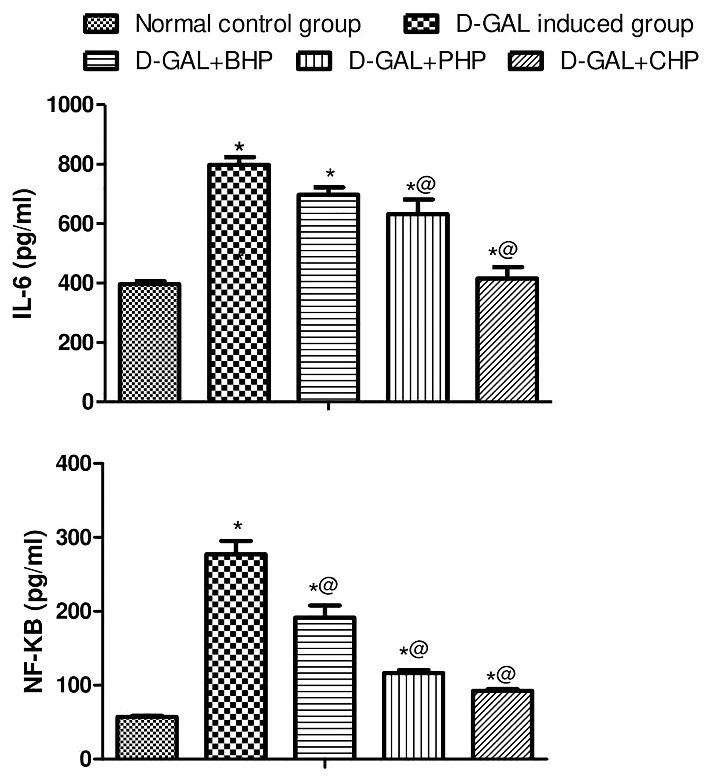
Effect of *H. pluvialis* on Il-6 (a) and NF-KB (b) in D-Gal induced hepatic aging in albino rats *: significantly different from normal control group at P≤0.05 @: significantly different from D-Gal- control at P≤0.05

Oral treatment of D-Gal-injected rats by PHP (30 mg/kg) or CHP (30 mg/kg) showed a decrease in serum ALT and AST with no significant effect of the biomass on the ALT and AST. Moreover, treatment with *H. pluvialis* showed a decrease in hepatic TG and TC content. Treatment of D-Gal-injected rats by BHP, PHP or CHP elevated hepatic level of catalase, GST and MPO as shown in Table 1. Likewise, BHP (450 mg/kg), PHP (30 mg/kg) or CHP (30 mg/kg) elevated hepatic levels of Nrf2 dramatically as shown in Figure 3. Treatment of hepatic injured rats with BHP, PHP or CHP showed reduction in hepatic IL-6 and NF-KB as shown in Figure 4.

**Table 1:** Effect of *H. pluvialis* on hepatic biochemical and oxidative stress parameters on D-Gal induced hepatic aging in albino rats

**Groups**	**Parameters**						
	**Serum ALT** **(U/ml)**	**Serum AST** **(U/ml)**	**Hepatic TG** **(mg/g tissue)**	**Hepatic TC** **(mg/g tissue)**	**Hepatic Catalase** **(U/g tissue)**	**Hepatic GST** **(U/g tissue)**	**Hepatic MPO** **(U/g tissue)**
**Normal**	55.22±1.38	86.35±4.34	149.88±6.13	28.12±3.01	0.81±0.09	6.53±0.12	4.13±0.31
**D-GAL**	139.76±5.03*	225.04±19.11*	709.90±36.61*	72.70±4.3*	0.23±0.023*	3.32±0.18*	12.34±1.01*
**D-GAL+BHP**	122.93±7.56*	179.00±12.92*	534.22±52.35*^@^	56.35±4.44*^@^	0.62±0.08^@^	5.65±0.42^@^	9.63±0.96*
**D-GAL+PHP**	101.81±5.77*^@^	174.58±1.74*^@^	523.81±51.31*^@^	55.12±3.04*^@^	0.63±0.07^@^	5.77±0.22^@^	8.58±0.72
**D-GAL+CHP**	94.32±6.01*^@^	164.37±13.52*^@^	498.39±43.15*^@^	48.05±4.19*^@^	0.67±0.07^@^	5.99±0.12^@^	4.36±2.03^@^

*: significantly different from normal control group at P≤0.05. @: significantly different from D-Gal-treated group at P≤0.05

However, the carotenoid fraction of *H. pluvialis* showed more prominent effect than the biomass and the polar fraction. This is attributed to the high content of ATX which is estimated to be 28.7 mg/100g in the free form and 21.2 mg/100g conjugated in the form of esters with fatty acids. The carotenoid fraction also contained 15.5 mg/100g β-carotene which is also known for its high antioxidant capability.

Astaxanthin-rich *H. pluvialis* increased the endogenous antioxidants and decreased the oxidative stress via activating Nrf2 which is one of the most important transcription factors in regulating multiple antioxidants, and it binds to the antioxidant response elements.^23^ It also plays a critical role in the regulation of the cellular GSH homeostasis. It can neutralize free radicals and may reduce or even help to prevent some of the damage caused by ROS.^24^

The potency of ATX varies greatly according to the configuration of its chairal centers. It is known that ATX isolated from *H. pulivialis* is the all trans-isomer,^25^ that’s why a docking study was performed to define the affinity of all trans ATX towards the key regulatory factors believed to be involved in the modulatory effect of ATX on hepatic aging namely Nrf2, IL-6 and NF-KB. The high affinity between all trans-astaxanthin and Nrf2 as indicated by the negative energy of binding indicates the possible direct interaction. This interaction is likely to be achieved through the dissociation of the complex of Nrf2/ keap liberating the free Nrf2 to react with antioxidant response elements and increase the expression of endogenous antioxidants allowing it to face oxidative stress state. On the contrary, docking of the 9-cis isomer of ATX, which is the synthetic form, resulted in a dramatic increase in the estimated energy of binding indicating very poor affinity and consequently negligible interaction between 9-cis ATX and Nrf2. That explains the vast potency variability between natural ATX and synthetic one.

**Figure 5:**
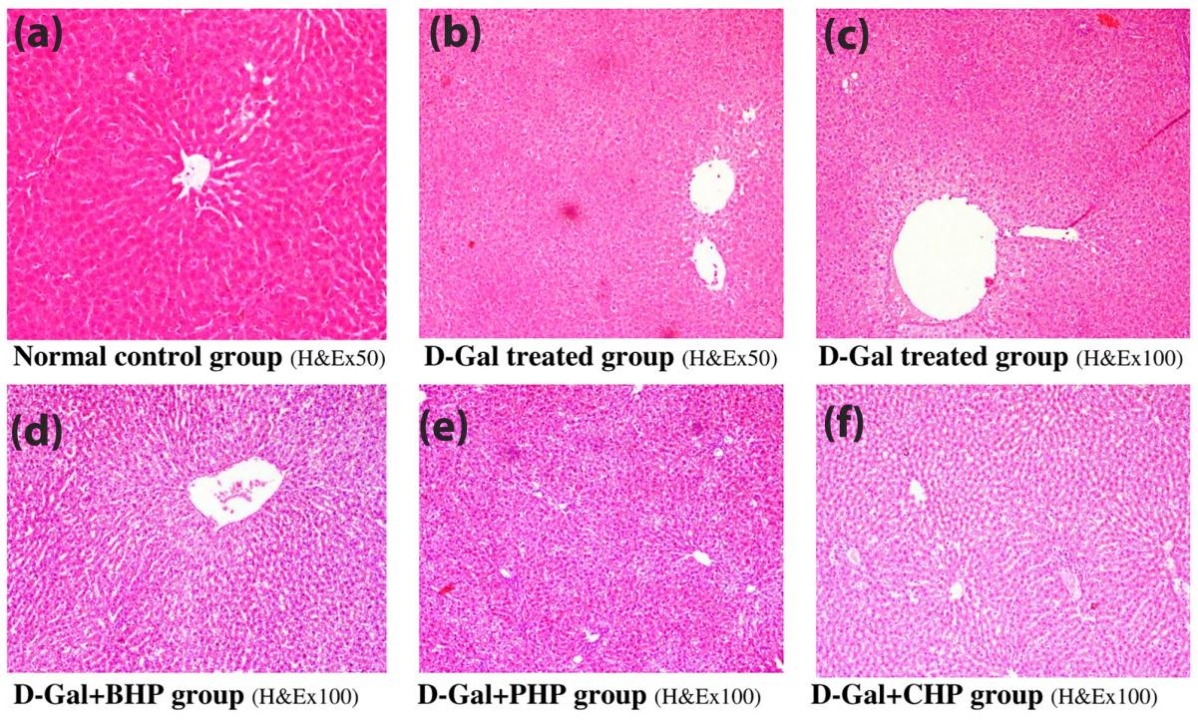
Effect of H. pluvialis on hepatic histopathological changes on D-Gal induced hepatic aging in albino rats

Moreover, aging showed dramatic changes in the histopathological picture showing dilatation of the central vein and congested inflammatory cellular infiltration at various areas as well as obliterated sinusoids and necrosis and cytoplasmic vacuolations of hepatocytes. *H. pluvialis* biomass and its fractions showed mild cytoplasmic vacuolations all over the three hepatic zones with dilated congested central vein with fatty infiltration of the liver and mild inflammatory cellular infiltration as shown in Figure 5.

## Conclusion

From all the previous results we can conclude that ATX-rich *H. pluvialis* has ameliorated the hepatic changes associated aging by enhancement of the endogenous antioxidant capacity due to its modulatory role of the Nrf2/Keap pathway and by inhibition of inflammatory mediators.

## Acknowledgments

The authors are thankful to Dr. Rofanda M. Bakeer, Department of Pathology, National Research Centre, Egypt, for the kind help in histopathology.

## Ethical Issues

The experiment was conducted in accordance with ethical procedures was approved by the National Research Centre (Dokki, Giza-Egypt)—Medical Research Ethics Committee for the use of animal subjects.

## Conflict of Interest

The authors have declared no conflict of interest.

## References

[1] Abdel-Misih SR, Bloomston M (2010). Liver anatomy. Surg Clin North Am.

[2] Kim IH, Kisseleva T, Brenner DA (2015). Aging and liver disease. Curr Opin Gastroenterol.

[3] Fisher-Wellman K, Bell HK, Bloomer RJ (2009). Oxidative stress and antioxidant defense mechanisms linked to exercise during cardiopulmonary and metabolic disorders. Oxid Med Cell Longev.

[4] Gemma C, Vila J, Bachstetter A, Bickford PC. Oxidative Stress and the Aging Brain: From Theory to Prevention. In: Riddle DR, editor. Brain Aging: Models, Methods, and Mechanisms. Boca Raton (FL): CRC Press/Taylor & Francis; 2007. 21204345

[5] Li S, Tan HY, Wang N, Zhang ZJ, Lao L, Wong CW (2015). The Role of Oxidative Stress and Antioxidants in Liver Diseases. Int J Mol Sci.

[6] Chambel SS, Santos-Goncalves A, Duarte TL (2015). The Dual Role of Nrf2 in Nonalcoholic Fatty Liver Disease: Regulation of Antioxidant Defenses and Hepatic Lipid Metabolism. Biomed Res Int.

[7] Sykiotis GP, Habeos IG, Samuelson AV, Bohmann D (2011). The role of the antioxidant and longevity-promoting Nrf2 pathway in metabolic regulation. Curr Opin Clin Nutr Metab Care.

[8] Davalli P, Mitic T, Caporali A, Lauriola A, D'Arca D (2016). ROS, Cell Senescence, and Novel Molecular Mechanisms in Aging and Age-Related Diseases. Oxid Med Cell Longev.

[9] Shah MM, Liang Y, Cheng JJ, Daroch M (2016). Astaxanthin-Producing Green Microalga Haematococcus pluvialis: From Single Cell to High Value Commercial Products. Front Plant Sci.

[10] El-Baz FK, Aly HF, Khalil WKB, Ali GH, Hafiz NA, Saad SA (2017). Potential role of Haematococcus pluvialis against diabetes induced oxidative stress and inflammation in rats. Asian J Pharm Clin Res.

[11] Abdo MS, Ahmed E, Abo El-Enin S, El Din RS, El Diwani G, Ali G (2013). Growth Rate and Fatty Acids Profile of 19 Microalgal Strains Isolated from River Nile for Biodiesel Production. J Algal Biomass Utln.

[12] Bikadi Z, Hazai E (2009). Application of the PM6 semi-empirical method to modeling proteins enhances docking accuracy of AutoDock. J Cheminform.

[13] Morris G, Goodsell D, Halliday R, Huuey R, Belew R, Olsen A (1998). Automated docking using a Lamarckian genetic algorithm and an empirical binding free energy function. J Comput Chem.

[14] Solis F, Wets R (1981). Minimization by Random Search Techniques. Mathematic Operat Res.

[15] Reitman S, Frankel S (1957). A colorimetric method for the determination of serum glutamic oxalacetic and glutamic pyruvic transaminases. Am J Clin Pathol.

[16] Fossati P, Prencipe L (1982). Serum triglycerides determined colorimetrically with an enzyme that produces hydrogen peroxide. Clin Chem.

[17] Richmond W (1973). Preparation and properties of a cholesterol oxidase from Nocardia sp. and its application to the enzymatic assay of total cholesterol in serum. Clin Chem.

[18] Wei H, Frenkel K (1993). Relationship of oxidative events and DNA oxidation in SENCAR mice to in vivo promoting activity of phorbol ester-type tumor promoters. Carcinogenesis.

[19] Wilce MC, Parker MW (1994). Structure and function of glutathione S-transferases. Biochim Biophys Acta.

[20] Ferrari SL, Ahn-Luong L, Garnero P, Humphries SE, Greenspan SL (2003). Two promoter polymorphisms regulating interleukin-6 gene expression are associated with circulating levels of C-reactive protein and markers of bone resorption in postmenopausal women. J Clin Endocrinol Metab.

[21] El-Baz FK, Hussein RA, Mahmoud K, Abdo SM (2018). Cytotoxic activity of carotenoid rich fractions from Haematococcus pluvialis and Dunaliella salina microalgae and the identification of the phytoconstituents using LC-DAD/ESI-MS. Phytother Res.

[22] Parameshwaran K, Irwin MH, Steliou K, Pinkert CA (2010). D-galactose effectiveness in modeling aging and therapeutic antioxidant treatment in mice. Rejuvenation Res.

[23] Regnier P, Bastias J, Rodriguez-Ruiz V, Caballero-Casero N, Caballo C, Sicilia D (2015). Astaxanthin from Haematococcus pluvialis Prevents Oxidative Stress on Human Endothelial Cells without Toxicity. Mar Drugs.

[24] Kobayashi M (2000). In vivo antioxidant role of astaxanthin under oxidative stress in the green alga Haematococcus pluvialis. Appl Microbiol Biotechnol.

[25] Weihong S, Hong L, Yuxiu Z, Limin C, Kailiang L, Lihong X (2015). Separation, Purification, and Identification of (3S,3′S)-trans-Astaxanthin from Haematococcus pluvialis. Sep Sci Technol.

